# Supplementary material for: Correction: Ethanol Suppresses PGC-1α Expression by Interfering with the cAMP-CREB Pathway in Neuronal Cells
Source: PLoS One. 2014 Sep 10;9(9):e108118. doi: 10.1371/journal.pone.0108118 (PMC4160296; doi:10.1371/journal.pone.0108118)
Supplement: File S2 — Republished, corrected article (PDF) [file pone.0108118.s002.pdf]

PLOS | ONE

Ethanol Suppresses *PGC-1α* Expression by Interfering with the cAMP-CREB Pathway in Neuronal Cells

Zilong Liu[1,4], Yongping Liu[1], Rui Gao[1], Haixia Li[2], Tiffany Dunn[3], Ping Wu[3], Robert G. Smith[1], Partha S. Sarkar[1,3], Xiang Fang[1]*

1 Department of Neurology, University of Texas Medical Branch, Galveston, Texas, United States of America, **2** Department of Internal Medicine/Gastroenterology, University of Texas Medical Branch, Galveston, Texas, United States of America, **3** Department of Neuroscience & Cell Biology, University of Texas Medical Branch, Galveston, Texas, United States of America, **4** Department of Forensic Medicine, Tongji Medical College of Huazhong University of Science and Technology, Wuhan, China

Abstract

Alcohol intoxication results in neuronal apoptosis, neurodegeneration and manifest with impaired balance, loss of muscle coordination and behavioral changes. One of the early events of alcohol intoxication is mitochondrial (Mt) dysfunction and disruption of intracellular redox homeostasis. The mechanisms by which alcohol causes Mt dysfunction, disrupts cellular redox homeostasis and triggers neurodegeneration remains to be further investigated. Proliferator-activated receptor gamma co-activator 1-alpha (*PGC-1α*) plays critical roles in regulating Mt biogenesis and respiration, cellular antioxidant defense mechanism, and maintenance of neuronal integrity and function. In this study, we sought to investigate whether alcohol causes Mt dysfunction and triggers neurodegeneration by suppressing *PGC-1α* expression. We report that ethanol suppresses *PGC-1α* expression, and impairs mitochondrial function and enhances cellular toxicity in cultured neuronal cell line and also in human fetal brain neural stem cell-derived primary neurons. Moreover, we report that cells over-expressing exogenous *PGC-1α* or treated with Rolipram, a selective phosphodiesterase-4 inhibitor, ameliorate alcohol-induced cellular toxicity. Further analysis show that ethanol decreases steady-state intracellular cAMP levels, and thus depletes phosphorylation of cAMP-response element binding protein (p-CREB), the key transcription factor that regulates transcription of *PGC-1α gene*. Accordingly, we found *PGC-1α* promoter activity and transcription was dramatically repressed in neuronal cells when exposed to ethanol, suggesting that ethanol blunts cAMP→CREB signaling pathway to interfere with the transcription of *PGC-1α*. Ethanol-mediated decrease in *PGC-1α* activity results in the disruption of Mt respiration and function and higher cellular toxicity. This study might lead to potential therapeutic intervention to ameliorate alcohol-induced apoptosis and/or neurodegeneration by targeting *PGC-1α*.

Citation: Liu Z, Liu Y, Gao R, Li H, Dunn T, et al. (2014) Ethanol Suppresses *PGC-1α* Expression by Interfering with the cAMP-CREB Pathway in Neuronal Cells. PLoS ONE 9(8): e104247. doi:10.1371/journal.pone.0104247

Editor: Yidong Bai, University of Texas Health Science Center at San Antonio, United States of America

Received March 10, 2014; **Accepted** June 25, 2014; **Published** August 6, 2014

Copyright: © 2014 Liu et al. This is an open-access article distributed under the terms of the Creative Commons Attribution License, which permits unrestricted use, distribution, and reproduction in any medium, provided the original author and source are credited.

Data Availability: The authors confirm that all data underlying the findings are fully available without restriction. Data are available at Figshare with the DOI:http://dx.doi.org/10.6084/m9.figshare.1091383.

Funding: This study was supported by NINDS grant 1K02NS081000-01A1), and University of Texas Medical Branch (UTMB) start-up fund. The funders had no role in study design, data collection and analysis, decision to publish, or preparation of the manuscript.

Competing Interests: The authors have declared that no competing interests exist.

* Email: xiang.fang01@gmail.com

Introduction

Alcohol (ethanol) is a common psychoactive substance, which is causally correlated with more than 60 different medical conditions, particularly the central nervous system disorders and contributes to 4% of the global burden of disease [1]. It has been well-demonstrated that acute and chronic alcohol intoxication results in significant alterations of brain structure and function, and induces neuronal apoptosis and neurodegeneration [2–4]. Alcohol intoxication manifests clinically as impaired balance, loss of muscle coordination and behavioral changes [5,6].

One of the most important underlying mechanisms involved in ethanol-induced neuronal apoptosis and/or neurodegeneration is the disruption of intracellular redox homeostasis [7]. Alcohol increases the productions of intracellular reactive oxygen species (ROS) [8], which has been implicated in the etiology of many alcohol-induced pathological conditions. Mitochondria (Mt) are known to be an important metabolic source of ROS in most cells. Dysfunction of mitochondrial energy metabolism have been described following acute and chronic ethanol exposure, leading to reduced ATP production and exacerbated ROS generation, contributing to increased susceptibility of cellular apoptosis and/or death to oxidative stressors [8,9].

Peroxisome proliferator-activated receptor-γ co-activator 1α (*PGC-1α*) is a member of transcription co-activator family. It plays important roles in regulating cellular energy metabolism, Mt biogenesis/respiration, cellular antioxidant surveillance mechanism, and maintenance of neuronal integrity and function [10–12]. PGC-1α interacts with a variety of transcription factors that regulate Mt biogenesis, Mt respiration, glucose transport and metabolism, fatty acid oxidation and cellular anti-oxidant properties. For example, PGC-1α protects neuron from H_2O_2-induced injury by up-regulating transcription of several antioxidant genes [11]. Decreased activity of PGC-1α has been implicated in the pathogenesis of numerous neurodegenerative diseases such as Huntington disease, Alzheimer disease, and Parkinson diseases [11,13–15]. Furthermore, PGC-1α is also involved in many

metabolic and traumatic brain diseases [11,16–18]. Acute alcohol exposure has been shown to suppress *PGC-1α* expression resulting in reduced expression of PGC-1α target genes regulating ROS metabolism contributing to liver injury *in vivo* animal model [19]. Taken together, we hypothesized that PGC-1α might play an important role in alcohol-induced Mt dysfunction and neurodegeneration.

The purpose of the present study was to investigate whether alcohol inflicts cellular toxicity via suppressing *PGC-1α* expression and to delineate the mechanism by which alcohol suppress *PGC-1α* expression. We have used human neuroblastoma SH-SY5Y cells as well as human fetal brain neural stem cell-derived primary neurons to test our hypothesis.

Materials and Methods

Cell culture and alcohol treatment

Two types of human neural cells were used in the study: 1) Human neuroblastoma SH-SY5Y cells, and SH-SY5Y cells stably over-expressing *PGC-1α*. These cells were cultured in Dulbecco's modified Eagle's medium (DMEM, Cellgro, Manassas, VA, USA) supplemented with 1% nonessential amino acids, 10% fetal bovine serum (FBS, Cellgro), 100 U/mL penicillin, and 100 U/mL streptomycin at 37°C in 5% CO_2; 2) Human fetal brain neural stem cells (hNSC, line K048) originally provided by Dr. Clive Svendsen were cultured according to our previous published protocol [20]. Cells were expanded as neurospheres in growth medium containing a basic medium supplemented with 20 ng/ml recombinant human epidermal growth factor (EGF), 5 µg/ml heparin, 10 ng/ml recombinant human leukemia inhibitory factor (LIF), and 20 ng/ml recombinant human basic fibroblast growth factor (bFGF) at 37°C with 8.5% CO_2. The basic medium is serum-free and contained DMEM (high glucose, L-lutamine)/ Ham's F12 (3:1), 1.5% D-glucose, 15 mM HEPES, 25 µg/ml bovine insulin, 67 IU/ml/67 µg/ml penicillin/streptomycin, 100 µM putrescine, 100 µg/ml human transferrin, 30 nM sodium selenite, 20 nM progesterone and 2 mM L-glutamine. The cells were passaged every 10 to 11 days as previously described [21]. Half of the medium was changed to fresh medium after 2 days. For cell differentiation, 5 days after cultured in priming medium, 80% of the medium was changed to a B27 medium containing DMEM/Ham's F12 (3:1), 1.5% D-glucose, 15 mM HEPES, 67 IU/ml/67 µg/ml penicillin/streptomycin, and B27 (Invitrogen/GIBCO). Cells in B27 medium were cultured at 37°C with 5% CO_2.

Following an overnight culture in a serum free medium, the cells were incubated in medium containing different concentrations of alcohol at 37°C for 24 h. According to previous studies, 100, 300, 500, and 700 mM of ethanol (Aaper Alcohol and Chemical Co, Shelbyville, KY, USA) were used for our experiments [22–25]. To maintain and stabilize the ethanol concentration in the medium, all cultures were maintained in sealed containers [26].

Cell counting

Cell counting was performed to determine total amount of cells and to ensure that cells were not detached from the surface of the plates due to ethanol treatment [27]. Briefly, the culture medium was carefully aspirated from the well without disturbing the attached cells, and the aspirated medium was examined under microscope to ensure that no cell was floating in the medium or accidentally drawn off. Then 0.30 ml solution containing 0.125% trypsin and 0.05% EDTA and 0.20% Trypan blue in 0.01 M PBS was added to the well after medium removed to detach the cells. The number of both viable (Trypan blue-negative) and dead/dying (Trypan blue-positive) cells was counted using a hemocytometer.

MTS assay

CellTiter 96 AQueous One Solution Cell Proliferation Assay (Promega, USA), containing tetrazolium compound [3-(4,5 dimethylthiazol-2-yl)-5-(3-carboxymethoxyphenyl)-2-(4-sulfophenyl)-2H-tetrazolium, inner salt] (MTS) [28], was used to determine the cell viability and mitochondrial function. In brief, SH-SY5Y cells (1×10^5 cells/ml) were seeded in 96-well plates. Following overnight growth, at 37°C in humidified chamber containing 5% CO_2, the medium was removed and replaced with 100 µL medium containing 0, 100, 300, 500 or 700 mM ethanol. After 24 hours exposure to ethanol, 20 µL MTS solution (Sigma, St Louis, MO, USA) was added into each well and incubated for 2 to 4 hours. The absorbance was measured at 490 nm using a microplate reader (Fluostar OPTIMA, BMG, USA).

Cell Toxicity (LDH) assay

The release of the cytosolic enzyme LDH into the medium was measured to determine the cellular toxicity due to ethanol treatment according to the instruction provided by the manufacturer (Sigma-Aldrich) [29]. Briefly, the cells were exposed to 0, 100, 300, 500 or 700 mM ethanol with or without 30 µM Rolipram, (Sigma-Aldrich Co., St Louis, MO, USA) (a selective phosphodiesterase-4 inhibitor) in medium for 24 h or 300 mM ethanol for various times. After incubation, the medium was collected for the LDH measurement. The reaction mixtures were kept in dark incubation chamber for 30 min, and then the reactions was terminated by the addition of 1/10 volume of 1 N HCl into each well. Absorbance was measured at 490 nm with a multi plate reader (Fluostar OPTIMA).

Analysis of intracellular ATP levels

The cultured SH-SY5Y cells were treated with alcohol in 24-well plates, intracellular ATP was extracted from 100 µl of cell suspension by adding 1.5% trichloroacetic acid, according to the manufacturer's recommended protocol (ENLITEN ATP assay system bioluminescence detection kit, Promega, Madison, WI). The extracted ATP solution was neutralized by 0.75 M Tris acetate buffer (pH 7.75) and centrifuged for 5 min at 4,500×g. The resulting supernatant was used in ATP determination assays. The reaction was initiated by adding 15 µl of the extract to 100 µl of the luciferin-luciferase luminous reagent (Promega) in 96-well white plate. Light output from the reaction was measured with Fluostar OPTIMA well plate reader (BMG, Germany) in luminescence mode. The concentration of the intracellular ATP levels was normalized by total cell counts.

Transient transfections and luciferase assay

The SH-SY5Y cells were transfected with 0.8 µg/well of PGC-1α-delta-CRE-promoter-luciferase reporter plasmid (the CREB-binding sequences were deleted), PGC-1α-promoter-luciferase plasmid (encoding wild type *PGC-1α* promoter sequences upstream of luciferase reporter gene), PGL negative control plasmid or CMV-luciferase positive control plasmid respectively using Lipofectamine™ 2000 (Invitrogen, Carlsbad, CA). Cells transfected with the plasmid DNA mixtures were cultured for 48 h. After washing with PBS, the cells were lysed with the lysis buffer (Promega, USA). The cell lysates were mixed with Luciferase Assay Reagent (Promega, USA) in 96-well plate, and the light was measured using a 96-well microplate luminometer (Veritas, Promega, USA) [30].

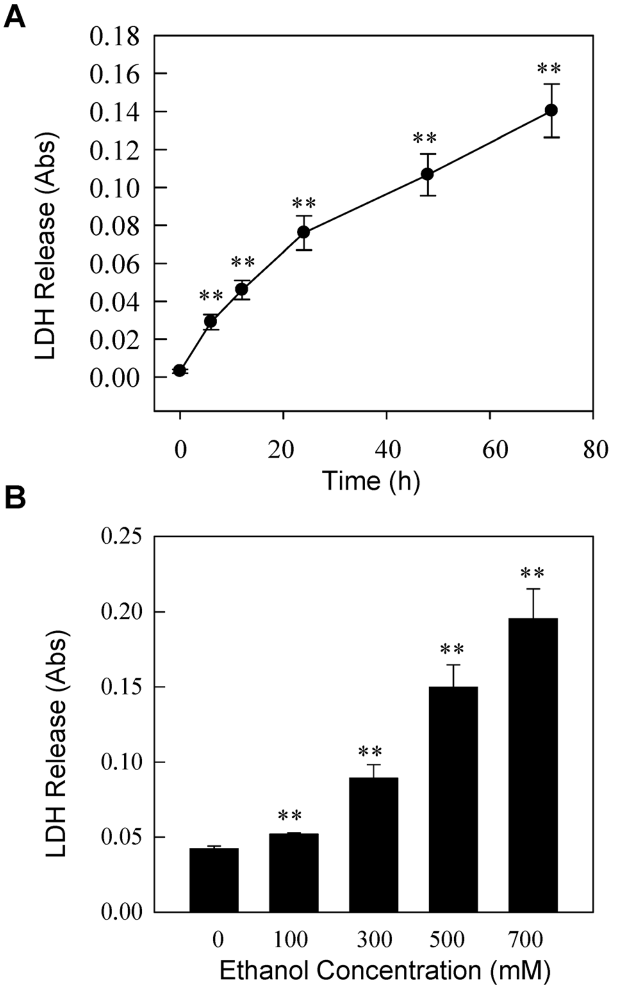

Figure 1. Time- and dose-dependent effects of ethanol on cellular toxicity in SH-SY5Y cells. The confluent SH-SY5Y cells were treated with 300 mM ethanol for various times (**Panel A**), or various concentrations of ethanol for 24 hours (**Panel B**). The cell culture medium was collected, and the LDH release was measured to assess ethanol-induced cellular toxicity. Mean ± SE, n = 4; ***P*<0.01 *vs.* control.
doi:10.1371/journal.pone.0104247.g001

Western blot analysis

Total proteins from cells were extracted and quantified with a BCA Protein Quantitative Analysis Kit (Pierce Biotechnology, Rockford, IL, USA). Proteins were separated on 8%–12% SDS-PAGE Gels (Life Technologies, Carlsbad, CA) and subsequently transferred to PVDF membranes (Bio-Rad, Hercules, CA, USA). The membranes were blocked with blocking buffer (0.1% Tween 20 in Tris-buffered saline, pH 7.4, containing 5% nonfat dried milk) at room temperature for 30 min and then incubated with antibodies against PGC-1α (1/1000, Santa Cruz Biotechnology, San Diego, CA, USA), PARIS (1/1000, NeuroMab, UC Davis, CA, USA), CREB (1/1000, Cell Signaling Technology), anti-phospho CREB (p-CREB, 1/1000, Cell Signaling Technology), and β-actin (1/5000, Abcam, Cambridge, MA, USA) in blocking buffer at 4°C overnight. After washing with PBST (PBS and 0.1% Tween 20) for 5 min for 3 times, the membrane was incubated with HRP-conjugated secondary antibody (1/5000) at room temperature for 2 h. The immunoreactive proteins were visualized by chemiluminescent reagent ECL (Pierce Biotechnology). Anti-β-actin antibody was used as loading control.

RNA extraction, cDNA synthesis and quantitative RT-PCR

Total RNA from SH-SY5Y cells before and after ethanol treatment was isolated using TRIzol reagent (Invitrogen, USA). Equal amount of total RNA (2 μg) were reversely transcribed using the SuperScript-II RT preamplication system (Invitrogen). Quantitative RT-PCR amplification (qRT-PCR) was carried out using specific primer pairs designed with Oligo Calculator and synthesized by IDT (MG, Brazil). Quantitative PCRs were carried out in an Applied-Biosystem StepOne Plus real-time cycler and done in quadruplicate. The PCR program was used as follows: 5 min at 95°C; 30 cycles of 45 sec at 94°C, 30 sec at 58°C and 30 sec at 72°C; and a final extension step of 10 min at 72°C. The following primer pairs were designed using the primer 5.0 software: PGC-1α forward 5′-CAAGGTCTCCAGGCAGTA-3′, reverse 5′-CACAGGTGGTAGGT-3′ and β-actin forward 5′-CAACTGGGACGATATGGAGAAG-3′, reverse 5′-TCTCCTTCTGCA TCCTGTCAG-3′. β-actin served as an internal control and for normalization. Expression of Mitochondrial uncoupling protein genes (UCP2 and UCP3), the PGC-1α target genes, were also measured by qRT-PCR. The UCP2 and UCP-3 primers were purchased from Qiagen (CA, USA).

Construction of plasmid DNA and transfection

The full length PGC-1α cDNA was PCR-amplified from plasmid pcDNA4-myc-PGC-1-alpha (kindly provided by Dr. Toren Finkel, Addgene plasmid 10974) [31] using appropriate primers and the full-length PGC-1α cDNA was sub-cloned in plasmid pCDNA3.1-hygro (Invitrogen, USA) to construct pcDNA-hygro-PGC-1α. The sequence integrity of PGC-1α cDNA in the expression plasmid was verified by sequencing. Plasmid DNA expressing PGC-1α was transfected into SH-SY5Y cells using Lipofectamine 2000 reagent (Invitrogen, USA) and the positive clones were selected with hygromycin (Invitrogen, USA). The effective expression of PGC-1α in the stable clones was verified by Western blot analysis using anti-PGC-1α antibody (Santa Cruz Biotechnology, USA). Plasmid DNA encoding 2 kb of the wild type PGC-1α promoter sequences (PGC-1α promoter 2 kb luciferase) and plasmid DNA wherein the CRE sequences in the PGC-1α promoter were deleted (PGC-1α promoter luciferase delta CRE) were kind gifts from Dr. Bruce Spiegelman (Addgene plasmids 8888 and 8887) [32].

Statistical analysis

All data are expressed as mean ± SE from at least three independent experiments. Comparisons of quantitative data were performed using one-way analysis of variance (ANOVA) followed by Tukey-test for multiple treated group. The difference between two treated groups was analyzed by student t-test. A *P*-value less than 0.05 was considered as significant.

Results

Ethanol-induced cellular toxicity in human neuroblastoma SH-SY5Y cells

To assess the extent to which alcohol causes cellular toxicity in neuronal cells, we treated human neuroblastoma SH-SY5Y cells with ethanol and assayed the alcohol-induced cellular toxicity using the Cell Toxicity Assay Kit (Sigma, USA). Previous studies showed that the high concentrations of alcohol are needed to produce cellular toxicities [22–25]. To assess cellular toxicity, the SH-SY5Y cells were incubated with cell culture medium containing 100, 300, 500 and 700 mM of ethanol for 24 hrs, or 300 mM ethanol for various times, and cell toxicity as well as cell viability was measured. The cellular viability was significantly

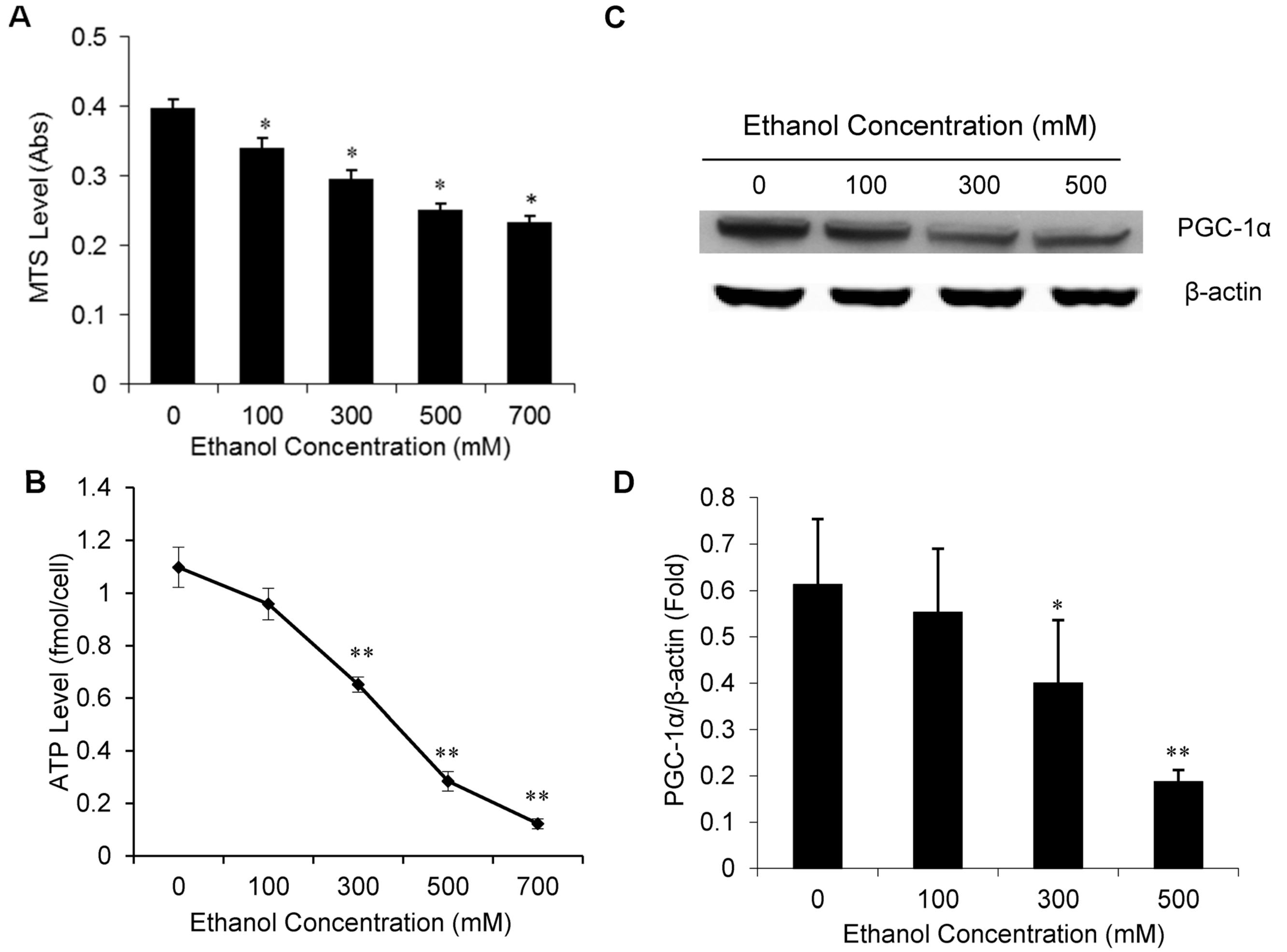

Figure 2. Concentration-dependent effect of ethanol on mitochondrial function, and expression of the *PGC-1α* in SH-SY5Y cells. The SH-SY5Y cells were treated with various concentration of ethanol for 24 hours, and MTS activity and intracellular ATP level were measured to assess mitochondrial function (**Panel A & B**). Total protein was isolated from the ethanol-treated and control cells and expression of *PGC-1α* was measured by Western blot analysis, and a representative Western blot is shown in Panel C. The protein density in each Western blot was quantitatively analyzed, and is shown in Panel D. Mean ± SE, n = 3, *$P<0.05$ *vs.* control, **$P<0.01$.
doi:10.1371/journal.pone.0104247.g002

decreased in all ethanol treatment groups compared to control group following 24 hrs of ethanol exposure (data not shown). Treatment of the SH-SY5Y cells with 300 mM ethanol produced a time-dependent and robust cytotoxicity as measured by LDH release by the ethanol-treated cells. Our data show that the amount of LDH release was increased by ~10-folds when treated with ethanol for 72 hrs (**Figure 1A**). We also found a dose-dependent increase in cell toxicity (LDH release) when cells were exposed to increasing alcohol concentrations. Compared to untreated control group, the LDH release from the ethanol-treated cells was increased by ~22% when treated with 100 mM ($P = 0.009$ *vs.* control), ~110% when treated with 300 mM ($P = 0.002$ *vs.* control), ~254% when treated with 500 mM ($P = 0.0004$ *vs.* control) and ~362.0% when treated with 700 mM ($P = 0.0002$ *vs.* control) of ethanol (**Figure 1B**). Consistent with the LDH release data, the cell viability was also decreased by 86% with 100 mM ($P = 0.02$ *vs.* control), 75% with 300 mM ($P = 0.0002$ *vs.* control), 63% with 500 mM ($P<0.0001$) and 59% with 700 mM ($P<0.0001$) ethanol treatment compared with the control groups.

Ethanol suppresses *PGC-1α* expression and blunts mitochondrial function

We next investigated the possible mechanism by which ethanol causes cellular toxicity and impairs Mt function. First, we sought to test whether alcohol impairs Mt respiration and suppresses PGC-1α expression. To test this idea, we treated SH-SY5Y cells with varying concentrations of ethanol and assessed Mt function using MTS assay, a versatile method to measure Mt function [2]. MTS assay measures the activity of Mt NAD(P)H-dependent cellular oxidoreductase enzyme succinic dehydrogenase. As shown in **Figure 2A**, ethanol treatment of the SH-SY5Y cells significantly decreased the MTS absorbance in a concentration-dependent manner. The MTS absorbance was decreased at least one-third in presence of 500 mM ethanol (**Figure 2A**). Since one of the major Mt functions is ATP production, we measured intracellular ATP production in SH-SY5Y cells treated with various concentration of ethanol for 24 hrs. Similar to MTS measurement, ethanol also produced a dose-dependent inhibition in intracellular ATP production (**Figure 2B**). The ATP production by SH-SY5Y cells was significantly suppressed at least ~70% in the presence of 500 mM ethanol as compared with control group (**Figure 2B**).

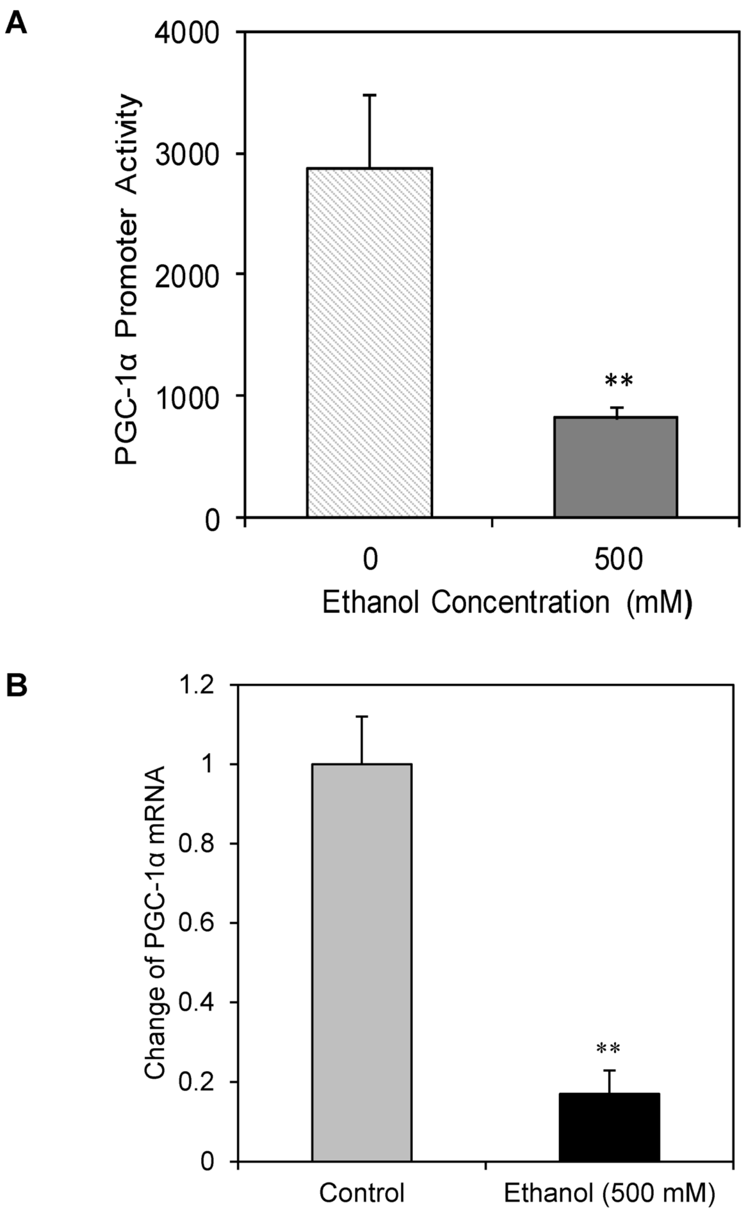

Figure 3. Effect of ethanol on PGC-1α promoter activity and *PGC-1α* expression in SH-SY5Y cells. The SH-SY5Y cells were co-transfected with *PGC-1α* promoter plasmid and luciferase-expression plasmids, and after 24 hours the cells were treated with various concentrations of ethanol for 24 hours. The ethanol-treated and control cells were lysed, and luciferase activity was measured (Panel A). In a separated experiment, total RNA was extracted from the ethanol-treated (500 mM) and control cells, and the level of PGC-1α mRNA was measured by real time quantitative RT-PCR (Panel B). Mean ± SE, n = 4, $^{**}P<0.01$ *vs.* Control.
doi:10.1371/journal.pone.0104247.g003

Since *PGC-1α* is a key regulator of the Mt biogenesis and function [10–12], we tested whether ethanol suppresses expression of *PGC-1α*. To test this idea, the SH-SY5Y cells were incubated with various concentrations of ethanol for 24 hours, and the steady-state levels of *PGC-1α* were measured by Western blot using a specific antibody against human PGC-1α. The Western blot data showed that ethanol decreased levels of PGC-1α protein in a dose-dependent manner (**Figure 2C**). The protein density analysis indicated that 500 mM and 300 mM ethanol decreased expression of *PGC-1α* protein by 66% ($P = 0.006$) and 26% ($P = 0.03$), respectively when the SH-SY5Y cells were treated with ethanol for 24 hrs (**Figure 2D**). Furthermore, we also tested whether ethanol affects expression of PGC-1α target genes. Expression of the *UCP-2* and *UCP-3* mRNA in the SH-SY5Y cells treated with or without ethanol was analyzed by qRT-PCR. We found that ethanol decreased expression of the UCP-2 and UCP-3 by ~70% when the cells were treated with 500 mM ethanol for 24 hrs.

Ethanol reduces *PGC-1α* levels by repressing transcription of *PGC-1α*

To determine whether ethanol suppresses expression of *PGC-1α* at the transcriptional level, we first determined the effects of ethanol on *PGC-1α* promoter activity in the ethanol-treated cells. The PGC-1α promoter reporter plasmid was constructed as described in the methods and materials section. Our results showed that treatment of cells with 500 mM ethanol inhibited

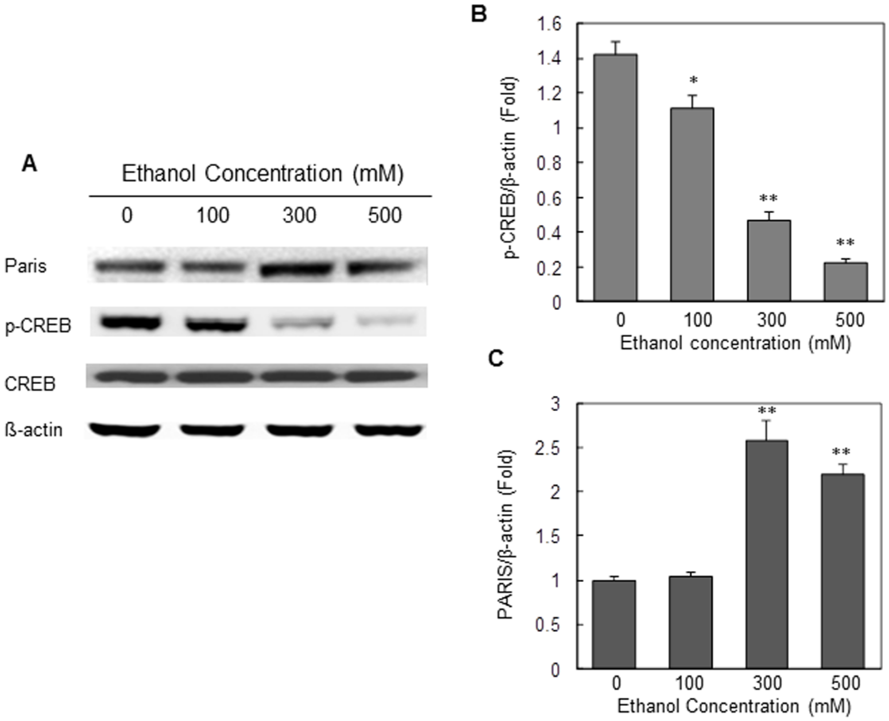

Figure 4. Dose-dependent effect of ethanol on the expression of CREB, P-CREB and PARIS in SH-SY5Y cells. The SH-SY5Y cells were treated with various concentrations of ethanol for 48 hours. After the ethanol treatment, the cells were harvested and the cell lysate was analyzed to determine expression of total CREB, p-CREB and PARIS by Western blot analysis. β-actin was used for loading control. The protein density in each Western blot was quantitatively analyzed, and is shown in Panels B and C. Mean ± SE, n = 3, *$P<0.05$, **$P<0.01$ *vs.* control.
doi:10.1371/journal.pone.0104247.g004

PGC-1α promoter activity by 83% when cells were incubated with ethanol for 24 hours (**Figure 3A**). Consistent with this data, we also found ethanol suppressed *PGC-1α* mRNA levels by ~80% when the cells were exposed to 500 mM ethanol (**Figure 3B**).

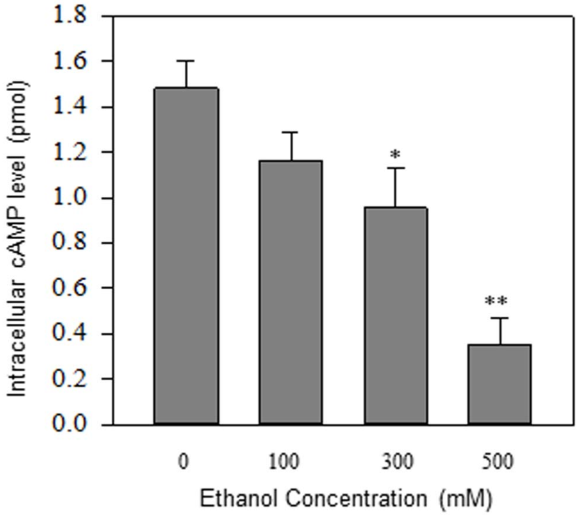

Figure 5. Dose-dependent effects of ethanol on intracellular cAMP level in SH-SY5Y cells. The confluent SH-SY5Y cells were treated with various concentrations of ethanol for 24 hours. The cells were corrected and lysed, and cellular cAMP levels were measured by cAMP analysis kit. Mean ± SE, n = 4. *$P<0.05$ vs. control, and **$P<0.01$ *vs.* control.
doi:10.1371/journal.pone.0104247.g005

These results suggested that the ethanol suppress *PGC-1α* expression by inhibiting its transcription.

Ethanol suppresses *PGC-1α* transcription by reducing phosphorylation of CREB

Several transcriptional factors have been identified that regulate *PGC-1α* transcription. For example, phosphorylated cAMP-responsive element binding protein (p-CREB) has been shown to bind the CRE consensus sequences proximal to the *PGC-1α* promoter and regulate *PGC-1α* transcription [33,34]. Therefore, we tested whether ethanol suppresses transcription of *PGC-1α* by repressing phosphorylation of CREB. To test this idea, the SH-SY5Y cells were incubated with cell culture medium containing various concentrations of ethanol for 24 hrs. After incubating the cells with ethanol, cells were harvested and the cell extracts were analyzed to assess the expression levels of CREB and p-CREB by Western blot analysis using specific antibodies against CREB and p-CREB. As shown in **Figure 4A**, ethanol treatment significantly decreased the steady-state levels of p-CREB, while levels of total CREB remained unchanged after ethanol treatment for 24 hrs. The protein density analysis indicated that level of p-CREB was significantly suppressed by ethanol in a dose-dependent manner (**Figure 4B**).

To further investigate the possible role of CREB pathway in the regulation of ethanol-mediated suppression of PGC1-α, we tested whether ethanol inhibits the promoter activity of PGC-1α. We used two plasmids for the promoter activity assay; plasmid PGC-1-alpha-promoter-2 kb-luciferase [26], where the wild type PGC-1α promoter sequences (~2 kb) was cloned upstream of reporter luciferase; and plasmid PGC-1-alpha-promoter-luciferase-delta CRE, wherein the CREB-binding CRE sequences were deleted

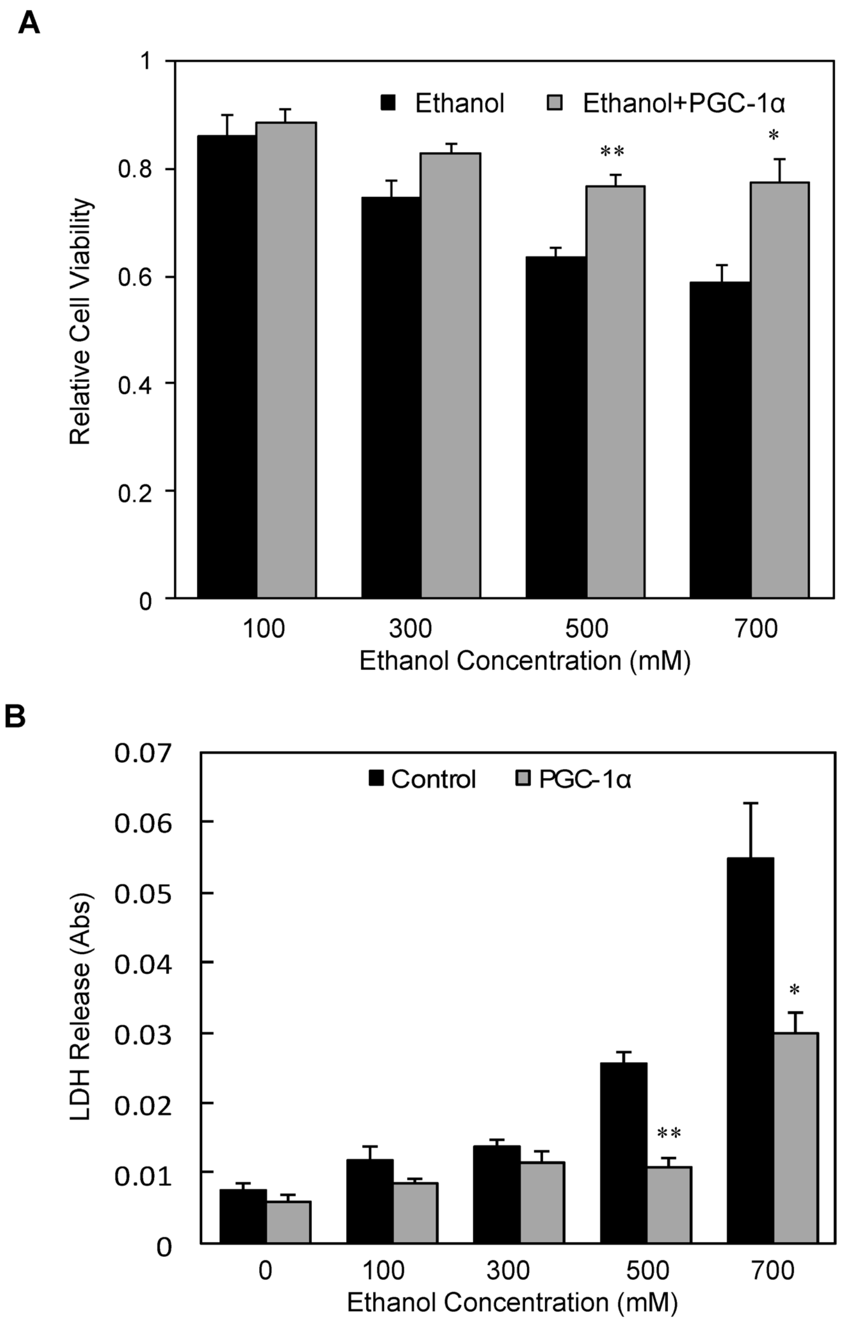

Figure 6. Over-expression of PGC-1α protect SH-SY5Y cells from ethanol-induced cellular toxicity. The SH-SY5Y cell line constitutively over-expressing PGC-1α was treated with various concentrations of alcohol and cellular toxicity (relative cell viability: panel A; LDH release: panel B) was measured as described in Figure 1. Mean ± SE, n = 4, *$P<0.05$. **$P<0.01$ *vs.* control.
doi:10.1371/journal.pone.0104247.g006

[32]. The delta-CRE-PGC-1α promoter plasmid and PGC-1α-promoter-2 kb-luciferase control plasmid, was transfected into alcohol-treated and untreated SH-SY5Y cells and luciferase activity was measured. We found that *PGC1-α* promoter-luciferase activity was ~70% decreased in cells transfected with wild type PGC-1α promoter plasmid; and about 40% decreased compared with the cells transfected with delta-CRE-*PGC-1α* promoter plasmid when the cells were treated with 500 mM ethanol for 24 hrs. This data suggests that CREB-binding sequences are important in alcohol-induced suppression of PGC-1α transcription, and indicates that cAMP→CREB signaling pathway might be affected by alcohol. To test this hypothesis, we investigated the possible mechanism by which ethanol decreases CREB phosphorylation. Since cAMP levels play an important role in regulating phosphorylation and activity of CREB, we tested whether ethanol decreases intracellular cAMP levels. As shown in **Figure 5**, our data showed that ethanol produced a dose-dependent decrease in the steady-state intracellular cAMP levels. We observed a ~70% reduction of intracellular cAMP when cells were treated with 500 mM ethanol compared with untreated control. These results suggest that alcohol suppresses CREB phosphorylation and expression of the *PGC-1α*, at least in part, by

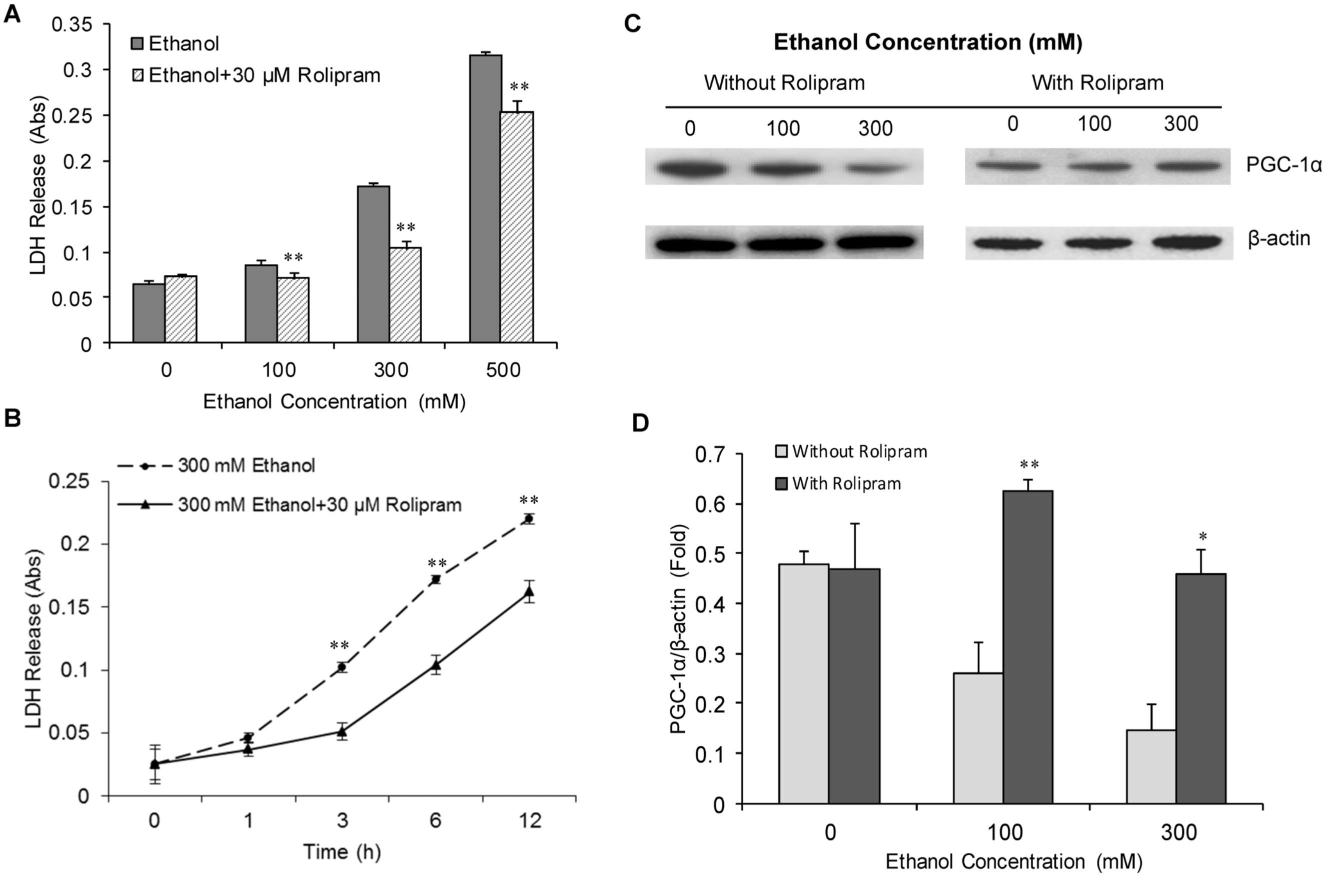

Figure 7. Effect of a PDE-4 inhibitor on ethanol-induced cytotoxicity and the expression of PGC-1α in SH-SY5Y cells. The confluent SH-SY5Y cells were pretreated with 30 μM Rolipram (a selective PDE-4 inhibitor) for 2 hrs, and then continuously treated with same concentration of Rolipram in the presence of various concentrations of alcohol for 24 hrs or 300 mM ethanol for different times. LDH release in the medium was measured, and the expression of PGC-1α was analyzed by Western-blot as described in Figure 2. Mean ± SE, n = 4. *$P<0.05$ *vs.* control, and **$P<0.01$ *vs.* control.
doi:10.1371/journal.pone.0104247.g007

decreasing intracellular cAMP levels and thus by interfering with the cAMP-CREB-dependent signaling pathway.

Moreover, recent studies have shown that the Zn-finger transcriptional repressor protein ZNF746, also known as PARIS plays an important role in regulating *PGC-1α* transcription [15]. This study shows that PARIS acts as transcriptional repressor which binds with the insulin response sequences (IRS) present in the PGC-1α promoter sequence and suppresses *PGC-1α* transcription. Since alcohol dramatically decreased PGC-1α expression, we therefore tested whether ethanol alters expression of PARIS in neuronal cells and contributes to the suppression of *PGC-1α* expression. The Western blot analysis of the alcohol-treated cells showed that ethanol indeed increased the expression of PARIS by 2 fold when cells were exposed to higher concentration of ethanol (300 mM) (**Figure 4A & C**). These data suggest that alcohol not only decreases cAMP levels and CREB activity but also stimulates PARIS expression to suppress PGC-1α expression.

Over-expression of PGC-1α ameliorated ethanol-induced cellular toxicity in SH-SY5Y cells

Since ethanol suppressed expression of *PGC-1α*, and PGC-1α has protective effects against oxidative stress-induced cellular injury [11], we next tested whether over-expression of PGC-1α protects against alcohol-mediated cellular toxicity. To test this idea, we treated a SH-SY5Y stable cell line ectopically expressing PGC-1α and control SH-SY5Y cells with various concentrations of ethanol for 24 hrs, and assessed the cell viability and cell toxicity (LDH activity) in the treated and control untreated cells. Our data show that the stable cells over-expressing *PGC-1α* showed significantly higher resistance to alcohol-induced toxicity (**Figure 6**). The cell viability was also significantly improved when cells over-expressing PGC-1α were treated with 500 mM and 700 mM ethanol ($P = 0.003$ and 0.02, respectively) (**Figure 6A**). The protective effect of *PGC-1α* against ethanol-induced cytotoxicity was further demonstrated as the LDH release in the culture medium was significantly decreased in the group expressing *PGC-1α*. The cell toxicity (as assessed by LDH release) was decreased by about 65% when the cells expressing PGC-1α were treated with 500 mM and 700 mM ethanol as compared with the cells expressing control plasmid ($P = 0.0003$ and 0.02, respectively) (**Figure 6B**).

Protective effect of Rolipram on ethanol-induced cellular toxicity and suppression of PGC-1α expression in SH-SY5Y cells

Since ethanol suppresses expression of PGC-1α through a cAMP-pCREB dependent mechanism, then we tested whether increased intracellular cAMP can protect the cells from alcohol-induced cellular toxicity, and reverse alcohol-mediated suppres-

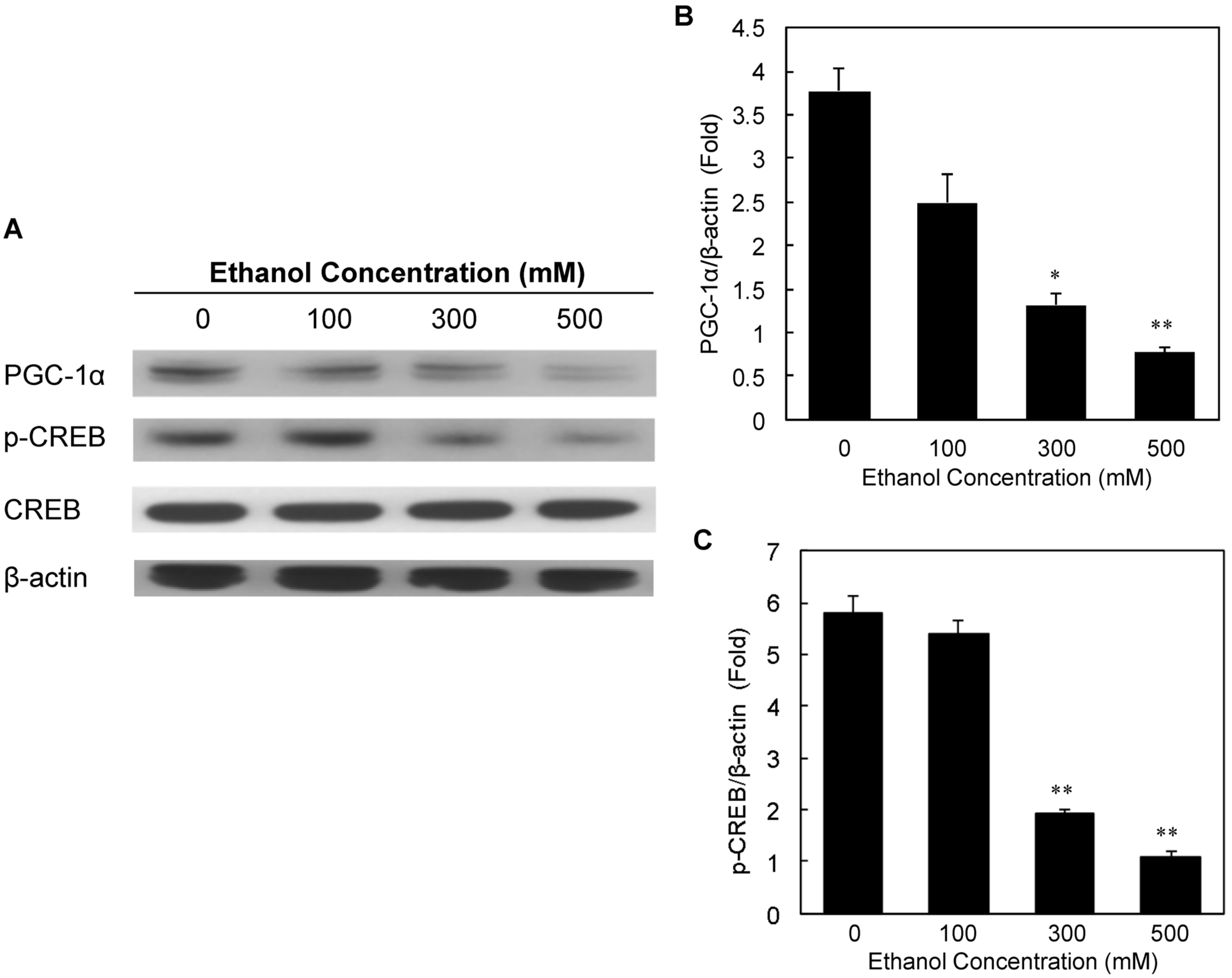

Figure 8. Dose-dependent effect of ethanol on the expression levels of PGC-1α, CREB, and p-CREB in human fetal brain neural stem cell-derived primary neurons. The primary neuronal cells were incubated with various concentrations of ethanol for 24 hrs, and after incubation with ethanol, the cells lysates were analyzed by Western blot to determine expression levels of PGC-1α, CREB, and p-CREB (Panel A). β-actin was used as loading control. The protein density in each Western blot was quantitatively analyzed, and is shown in Panels B and C. Mean ± SE, n = 3, **P*<0.05, ***P*<0.01 *vs.* control.
doi:10.1371/journal.pone.0104247.g008

sion of PGC-1α expression using a pharmacological approach. Rolipram, a selective phosphodiesterase (PDE)-4 inhibitor, has been shown to increase intracellular cAMP level in SH-SY5Y neuronal cells [35]. The cells were treated with or without 30 μM Rolipram in the presence of 300 mM ethanol for different times, or various concentrations of ethanol for 12 hrs. The amount of LDH in the medium and expression of PGC-1α protein in the cells were determined as described in the section of Methods. As shown in Figure 7, Rolipram provided significant protective effects against alcohol-induced cellular toxicity in a time- and dose-dependent manner as compared with ethanol alone group. 30 μM Rolipram decreased the LDH release by ~25 to 30% when the cells were exposed to 100 to 500 mM ethanol (**Figure 7A & B**). Furthermore, Rolipram also reversed ethanol-induced suppression of PGC-1α protein (**Figure 7C**). The protein density analysis indicated that expression of the PGC-1α was almost back to the baseline when the cells were treated with 30 μM Rolipram in the presence of 300 mM ethanol (**Figure 7D**).

Ethanol suppressed PGC-1α and decreased CREB phosphorylation in human fetal brain neural stem cell (hNSC)-derived primary neurons

Since alcohol inhibits neurogenesis in a CREB dependent mechanism [2], we tested the effect of ethanol on PGC-1α expression in hNSC-derived primary neurons. Similar to the data obtained using the SH-SY5Y cells, the ethanol treatment also produced a concentration-dependent toxicity in the hNSC-derived primary neurons (data not shown). We next tested whether ethanol suppressed expression of *PGC-1α* in the primary neurons. As shown in **Figure 8**, ethanol significantly inhibited the expression of *PGC-1α* in a dose-dependent manner (**Figure 8A & B**). Similarly, ethanol also suppressed phosphorylation of CREB, whereas total CREB levels remained unaltered upon ethanol treatment (**Figure 8A & C**). These data strongly support our hypothesis that alcohol can suppress PGC-1α transcription via interfering with the cAMP-CREB signaling pathway.

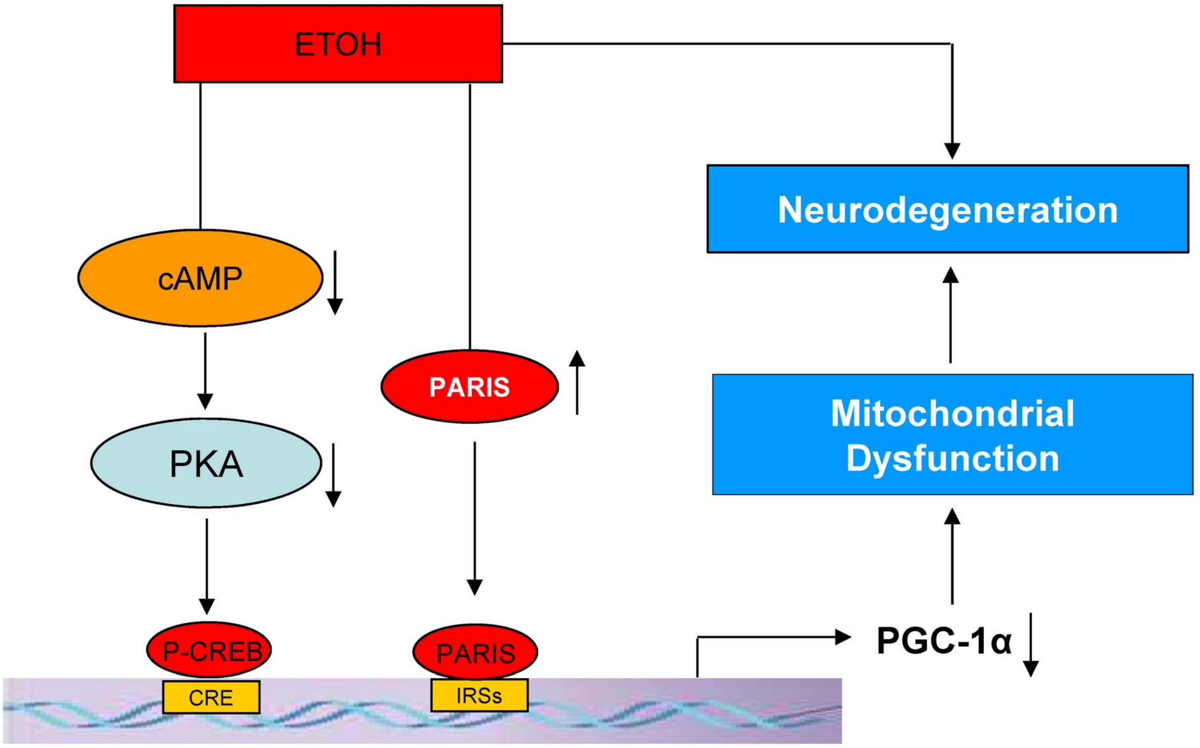

Figure 9. The proposed mechanisms for ethanol-mediated regulation of PGC-1α expression and neurodegeneration.
doi:10.1371/journal.pone.0104247.g009

Discussion

In the present study, we report that ethanol 1) produces a dose- and time-dependent cellular toxicity as well as blunts Mt respiration; 2) interferes with the PGC-1α promoter activity and thus suppresses expression of *PGC-1α*; 3) suppresses expression of the PGC-1α target genes UCP2 and UCP3; 4) diminishes CREB phosphorylation via reducing intracellular cAMP levels; 5) increases expression of Zn-finger protein Paris in SH-SY5Y cells; 6) decreases expression of PGC-1α and phosphorylation of CREB in hNSC-derived primary neurons. We also find that 1) over-expression of PGC-1α protects neuronal cells from alcohol-induced cell damage; 2) inhibition of PDE-4 by Rolipram protects ethanol-induced cellular toxicity and reverses ethanol-induced suppression of PGC-1α expression.

Mt dysfunction and disruption of intracellular redox homeostasis play important roles in alcohol-induced neurodegeneration [2]. Previous studies have shown that alcohol increases intracellular ROS level, produces mitochondrial damage which provokes mitochondrial-mediated apoptosis, and neuron death [36,37]. It is well-demonstrated that disruption of intracellular redox status causes impairment of function of proteins, lipids, nucleic acids, and triggers cascades of ROS-dependent signaling that activates programmed cell death [38]. Various antioxidants/ROS scavengers have been found to provide protective effects against alcohol-induced neural cell damage in *in vivo* and *in vitro* models [7,37,39–41]. Our unpublished data also showed that ethanol increased intracellular ROS levels, and NADPH oxidase-dependent superoxide production in SH-SY5Y cells (unpublished observation). Our present findings show that alcohol dramatically impairs Mt function (**Figure 2**) in SH-SY5Y cells. Considering a crucial role of *PGC-1α* in the regulation and maintenance of intracellular redox status and mitochondrial function, we tested the effects of ethanol on the expression of PGC-1α. Our data clearly show that ethanol decreases the expression of *PGC-1α* in a dose-dependent manner via suppressing *PGC-1α* promoter activity. These results suggest that ethanol regulates expression of *PGC-1α* at the transcriptional level. Consistent with our observation, alcohol has been shown to suppress expression of PGC-1α in H4-IIE cells and hepatic tissues [16]. Many PGC-1α target genes that are involved in regulation of mitochondrial biogenesis, muscle integrity, ROS detoxification, fatty acid metabolism, and insulin sensitivity have been identified. UCPs are those PGC-1α target genes that regulate mitochondrial transmembrane proton leakage, and ATP synthesis. We also found that the expression of UCP-2, and UCP-3 genes was down-regulated when the SH-SY5Y cells were treated with ethanol.

Several signaling pathways have been implicated in the regulation of *PGC-1α* transcription, e.g., CaMK, calcineurin A, PKC, and cAMP-CREB pathways [42–45]. cAMP is a second messenger derived from adenosine triphosphate (ATP) by a reaction catalyzed by adenylyl cyclase. Although our data clearly show that alcohol decreases steady-state cAMP levels, and we are yet to establish whether alcohol decreases cAMP levels by mitigating activity of adenylyl cyclase. It is also possible that alcohol might stimulate activity of phosphodiesterase to catalyze degradation of cAMP and thus reduces steady-state cAMP levels. Clearly additional experiments are necessary to further establish how alcohol decreases cAMP levels. Activation of the cAMP-CREB pathway results in the phosphorylation of CREB (p-CREB), and p-CREB binds the CREB-response element (CRE), located proximal to the *PGC-1α* promoter, and activates its transcription [46]. It has been reported that p-CREB stimulates the expression of *PGC-1α* by interacting with the consensus CRE sequences in the PGC-1α promoter region in neuronal cells [13]. Recent study revealed a suppressive effect of alcohol on CREB

activity, which might be involved in ethanol-induced cell damage [47]. In the present study, we also found that ethanol produced a dose-dependent suppression of phosphorylation of CREB in SH-SY5Y neuronal cells; whereas total CREB levels remained unaltered upon alcohol treatment. Furthermore, using mutated ΔCRE binding site at PGC-1α promoter, inhibitory effect of ethanol on PGC-1α promoter activity was significantly decreased suggesting that suppression of PGC-1α expression by alcohol is at least partly mediated via a CREB-dependent pathway.

The fact that deletion of CRE binding sequences proximal to the PGC-1α promoter sequences did not completely restore the inhibitory effect of ethanol on PGC-1α promoter activity suggests that additional transcriptional regulatory factors might also play an important role in alcohol-mediated suppression of *PGC-1α* transcription. Recently, the Zn-finger protein PARIS, also known as Znf746 has been identified which suppresses transcription of *PGC-1α* by binding to the insulin response sequence (IRS) present in the proximal region of the PGC-1α promoter sequences [15,48]. The activity of both human and mouse PGC-1α promoters is markedly decreased by PARIS, suggesting that the transcriptional repressor PARIS might play an important role in neurodegenerative disorders [15,49]. Shin et al [15] reported that PARIS is differentially expressed in the brain and is localized to neurons, the over-expression of PARIS leads to the loss of neurons mediated *via* repression of PGC-1α. Thus, we tested whether increased PARIS expression might also contribute to the ethanol-mediated expression of PGC-1α. When SH-SY5Y cells were treated with ethanol, expression of PARIS was significantly increased, concurrently with the decrease in PGC-1α expression. However, it is not clear how ethanol stimulates PARIS expression, and we are currently investigating the possible mechanisms by which ethanol increases PARIS expression. Our present findings thus suggest that ethanol suppresses transcription of *PGC-1α* by interfering with multiple but independent signaling pathways.

Previous studies have suggested that PGC-1α has neuroprotective effects against oxidative stress [11,19]. Since ethanol suppressed PGC-1α expression, we determined whether over-expression of PGC-1α can protect the neuronal cells from ethanol-induced damage. Our findings showed that over-expression of PGC-1α significantly prevented ethanol-mediated cell cytotoxicity as measured by cell viability and LDH release by the ethanol-treated SH-SY5Y cells. Consistent with the previous findings [11,19], we found that over-expression of PGC-1α also decreased the level of intracellular ROS when the cells were treated with ethanol (unpublished observations). It is likely that the antioxidant properties of PGC-1α might contribute to its anti-apoptotic and neuroprotective actions. However, additional effects such as the prevention of alcohol-induced dysfunction of mitochondrial energy metabolism [10–12] that can potentially contribute to the neuroprotective actions of PGC-1α still need to be further investigated.

Since PGC-1α is regulated by a cAMP-CREB dependent signaling pathway in ethanol-treated neuronal cells, an alternative approach is to determine whether inhibition of cAMP decomposition can protect the neuronal cells from ethanol-induced cellular damage, and reverse ethanol-mediated suppression of PGC-1α. Intracellular cAMP is further metabolized by a process catalyzed by PDE in most mammalian cells. Rolipram, a selective PDE-4 inhibitor, has been shown to increase intracellular cAMP in SH-SY5Y cells, and improve survival of spiral ganglion neurons [35,50]. Furthermore, PDE-4 inhibitors also increase the survival of dopaminergic neurons in primary cell culture [51]. We found that elevated intracellular cAMP by inhibiting PDE-4 can diminish ethanol-mediated suppression of PGC-1α expression, and therefore, protect the neuronal cells from ethanol-induced cell damage. These results further support our hypothesis that PGC-1α plays an important role in alcohol-induced pathogenesis in neuronal cells.

Furthermore, we found that ethanol suppressed expression of PGC-1α as well as reduced phosphorylation of CREB in hNSC-derived primary neuronal cells. Altered levels of CREB phosphorylation is implicated in neurodegeneration and neurogenesis during alcohol intoxication and abstinence. For example, regeneration of brain is related to increased CREB transcription, increased neurogenesis and cell genesis during alcohol abstinence [2]. Since mitochondrial dysfunction is involved in many neurodegenerative diseases and aging [52], and PGC-1α is a key regulator of mitochondrial function, our findings suggest that CREB target *PGC-1α* might play an important role in alcohol-induced process of neurodegeneration.

In conclusion, our study clearly shows that alcohol suppresses transcription of *PGC-1α* in both SH-SY5Y cells as well as in hNSC-derived primary neurons. Alcohol-induced suppression of PGC-1α might contribute to alcohol-mediated cell damage, and neurodegeneration that are commonly associated with alcohol overuse and alcoholism. Suppression of PGC-1α transcription by ethanol is mediated by interfering with the CREB and PARIS signaling pathways in these neurons. The proposed mechanisms for ethanol-mediated regulation of PGC-1α expression and neurodegeneration are illustrated in **Figure 9**. The fact that over-expression of PGC-1α has protective effects against alcohol-induced neuronal damage suggests that modulation of PGC-1α expression can be a novel therapeutic approach for the treatment of alcohol-induced apoptosis and/or neurodegeneration.

Acknowledgments

Authors thank Raymond Fang and Hongxia Pei for their technical assistances.

Author Contributions

Conceived and designed the experiments: XF ZLL PSS. Performed the experiments: ZLL YPL RG TD. Analyzed the data: XF PSS ZLL HXL. Contributed reagents/materials/analysis tools: PW RGS. Wrote the paper: XF ZLL PSS.

References

1. Room R, Babor T, Rehm J (2005) Alcohol and public health. Lancet 365: 519–530.
2. Crews FT, Nixon K (2009) Mechanisms of neurodegeneration and regeneration in alcoholism. Alcohol Alcohol 44: 115–127.
3. Dikranian K, Qin YQ, Labruyere J, Nemmers B, Olney JW (2005) Ethanol-induced neuroapoptosis in the developing rodent cerebellum and related brain stem structures. Brain Res Dev Brain Res 155: 1–13.
4. Garic-Stankovic A, Hernandez MR, Chiang PJ, Debelak-Kragtorp KA, Flentke GR, et al. (2005) Ethanol triggers neural crest apoptosis through the selective activation of a pertussis toxin-sensitive G protein and a phospholipase Cbeta-dependent Ca2+ transient. Alcohol Clin Exp Res 29: 1237–1246.
5. Echevarria DJ, Toms CN, Jouandot DJ (2011) Alcohol-induced behavior change in zebrafish models. Rev Neurosci 22: 85–93.
6. Patel M, Modig F, Magnusson M, Fransson PA (2010) Alcohol intoxication at 0.06 and 0.10% blood alcohol concentration changes segmental body movement coordination. Exp Brain Res 202: 431–443.
7. Antonio AM, Druse MJ (2008) Antioxidants prevent ethanol-associated apoptosis in fetal rhombencephalic neurons. Brain Res 1204: 16–23.
8. Bailey SM, Cunningham CC (1998) Acute and chronic ethanol increases reactive oxygen species generation and decreases viability in fresh, isolated rat hepatocytes. Hepatology 28: 1318–1326.

9. Ivester P, Lide MJ, Cunningham CC (1995) Effect of chronic ethanol consumption on the energy state and structural stability of periportal and perivenous hepatocytes. Arch Biochem Biophys 322: 14–21.
10. Liang H, Ward WF (2006) PGC-1alpha: a key regulator of energy metabolism. Adv Physiol Educ 30: 145–151.
11. St-Pierre J, Drori S, Uldry M, Silvaggi JM, Rhee J, et al. (2006) Suppression of reactive oxygen species and neurodegeneration by the PGC-1 transcriptional coactivators. Cell 127: 397–408.
12. Zhu L, Sun G, Zhang H, Zhang Y, Chen X, et al. (2009) PGC-1alpha is a key regulator of glucose-induced proliferation and migration in vascular smooth muscle cells. PLoS One 4: e4182.
13. Cui L, Jeong H, Borovecki F, Parkhurst CN, Tanese N, et al. (2006) Transcriptional repression of PGC-1alpha by mutant huntingtin leads to mitochondrial dysfunction and neurodegeneration. Cell 127: 59–69.
14. Qin W, Haroutunian V, Katsel P, Cardozo CP, Ho L, et al. (2009) PGC-1alpha expression decreases in the Alzheimer disease brain as a function of dementia. Arch Neurol 66: 352–361.
15. Shin JH, Ko HS, Kang H, Lee Y, Lee YI, et al. (2011) PARIS (ZNF746) repression of PGC-1alpha contributes to neurodegeneration in Parkinson's disease. Cell 144: 689–702.
16. Agrawal R, Tyagi E, Vergnes L, Reue K, Gomez-Pinilla F (2014) Coupling energy homeostasis with a mechanism to support plasticity in brain trauma. Biochim Biophys Acta 1842: 535–546.
17. Lin J, Wu PH, Tarr PT, Lindenberg KS, St-Pierre J, et al. (2004) Defects in adaptive energy metabolism with CNS-linked hyperactivity in PGC-1alpha null mice. Cell 119: 121–135.
18. Zheng B, Liao Z, Locascio JJ, Lesniak KA, Roderick SS, et al. (2010) PGC-1alpha, a potential therapeutic target for early intervention in Parkinson's disease. Sci Transl Med 2: 52ra73.
19. Chaung WW, Jacob A, Ji Y, Wang P (2008) Suppression of PGC-1alpha by Ethanol: Implications of Its Role in Alcohol Induced Liver Injury. Int J Clin Exp Med 1: 161–170.
20. Svendsen CN, ter Borg MG, Armstrong RJ, Rosser AE, Chandran S, et al. (1998) A new method for the rapid and long term growth of human neural precursor cells. J Neurosci Methods 85: 141–152.
21. Tarasenko YI, Yu Y, Jordan PM, Bottenstein J, Wu P (2004) Effect of growth factors on proliferation and phenotypic differentiation of human fetal neural stem cells. J Neurosci Res 78: 625–636.
22. Smith RA, Wubetu T (1991) Cytoplasmic changes in primary cultured adult mouse sensory neurons induced by ethanol and acetaldehyde treatments. Virchows Arch B Cell Pathol Incl Mol Pathol 61: 209–215.
23. Yan D, Dong J, Sulik KK, Chen SY (2010) Induction of the Nrf2-driven antioxidant response by tert-butylhydroquinone prevents ethanol-induced apoptosis in cranial neural crest cells. Biochem Pharmacol 80: 144–149.
24. Thibault C, Lai C, Wilke N, Duong B, Olive MF, et al. (2000) Expression profiling of neural cells reveals specific patterns of ethanol-responsive gene expression. Mol Pharmacol 58: 1593–1600.
25. Seiler AE, Ross BN, Rubin R (2001) Inhibition of insulin-like growth factor-1 receptor and IRS-2 signaling by ethanol in SH-SY5Y neuroblastoma cells. J Neurochem 76: 573–581.
26. Adickes ED, Mollner TJ, Lockwood SK (1988) Closed chamber system for delivery of ethanol to cell cultures. Alcohol Alcohol 23: 377–381.
27. Luo J, Miller MW (1997) Differential sensitivity of human neuroblastoma cell lines to ethanol: correlations with their proliferative responses to mitogenic growth factors and expression of growth factor receptors. Alcohol Clin Exp Res 21: 1186–1194.
28. Cory AH, Owen TC, Barltrop JA, Cory JG (1991) Use of an aqueous soluble tetrazolium/formazan assay for cell growth assays in culture. Cancer Commun 3: 207–212.
29. Legrand C, Bour JM, Jacob C, Capiaumont J, Martial A, et al. (1992) Lactate dehydrogenase (LDH) activity of the cultured eukaryotic cells as marker of the number of dead cells in the medium [corrected]. J Biotechnol 25: 231–243.
30. Sato O, Kuriki C, Fukui Y, Motojima K (2002) Dual promoter structure of mouse and human fatty acid translocase/CD36 genes and unique transcriptional activation by peroxisome proliferator-activated receptor alpha and gamma ligands. J Biol Chem 277: 15703–15711.
31. Ichida M, Nemoto S, Finkel T (2002) Identification of a specific molecular repressor of the peroxisome proliferator-activated receptor gamma Coactivator-1 alpha (PGC-1alpha). J Biol Chem 277: 50991–50995.
32. Handschin C, Rhee J, Lin J, Tarr PT, Spiegelman BM (2003) An autoregulatory loop controls peroxisome proliferator-activated receptor gamma coactivator 1alpha expression in muscle. Proc Natl Acad Sci U S A 100: 7111–7116.
33. Herzig S, Long F, Jhala US, Hedrick S, Quinn R, et al. (2001) CREB regulates hepatic gluconeogenesis through the coactivator PGC-1. Nature 413: 179–183.
34. Wu Z, Huang X, Feng Y, Handschin C, Gullicksen PS, et al. (2006) Transducer of regulated CREB-binding proteins (TORCs) induce PGC-1alpha transcription and mitochondrial biogenesis in muscle cells. Proc Natl Acad Sci U S A 103: 14379–14384.
35. Morgan AJ, Murray KJ, Challiss RA (1993) Comparison of the effect of isobutylmethylxanthine and phosphodiesterase-selective inhibitors on cAMP levels in SH-SY5Y neuroblastoma cells. Biochem Pharmacol 45: 2373–2380.
36. Yeo JE, Kim JH, Kang SK (2008) Selenium attenuates ROS-mediated apoptotic cell death of injured spinal cord through prevention of mitochondria dysfunction; in vitro and in vivo study. Cell Physiol Biochem 21: 225–238.
37. Zhang C, Tian X, Luo Y, Meng X (2011) Ginkgolide B attenuates ethanol-induced neurotoxicity through regulating NADPH oxidases. Toxicology 287: 124–130.
38. Aksenova MV, Aksenov MY, Mactutus CF, Booze RM (2005) Cell culture models of oxidative stress and injury in the central nervous system. Curr Neurovasc Res 2: 73–89.
39. Heaton MB, Madorsky I, Paiva M, Siler-Marsiglio KI (2004) Vitamin E amelioration of ethanol neurotoxicity involves modulation of apoptotis-related protein levels in neonatal rat cerebellar granule cells. Brain Res Dev Brain Res 150: 117–124.
40. Naseer MI, Lee HY, Kim MO (2010) Neuroprotective effect of vitamin C against the ethanol and nicotine modulation of GABA(B) receptor and PKA-alpha expression in prenatal rat brain. Synapse 64: 467–477.
41. Naseer MI, Ullah N, Ullah I, Koh PO, Lee HY, et al. (2010) Vitamin C protects against ethanol and PTZ-induced apoptotic neurodegeneration in prenatal rat hippocampal neurons. Synapse 65: 562–571.
42. Akimoto T, Pohnert SC, Li P, Zhang M, Gumbs C, et al. (2005) Exercise stimulates Pgc-1alpha transcription in skeletal muscle through activation of the p38 MAPK pathway. J Biol Chem 280: 19587–19593.
43. Fan M, Rhee J, St-Pierre J, Handschin C, Puigserver P, et al. (2004) Suppression of mitochondrial respiration through recruitment of p160 myb binding protein to PGC-1alpha: modulation by p38 MAPK. Genes Dev 18: 278–289.
44. Jager S, Handschin C, St-Pierre J, Spiegelman BM (2007) AMP-activated protein kinase (AMPK) action in skeletal muscle via direct phosphorylation of PGC-1alpha. Proc Natl Acad Sci U S A 104: 12017–12022.
45. Wu Z, Puigserver P, Andersson U, Zhang C, Adelmant G, et al. (1999) Mechanisms controlling mitochondrial biogenesis and respiration through the thermogenic coactivator PGC-1. Cell 98: 115–124.
46. Knutti D, Kralli A (2001) PGC-1, a versatile coactivator. Trends Endocrinol Metab 12: 360–365.
47. Sakai R, Ukai W, Sohma H, Hashimoto E, Yamamoto M, et al. (2005) Attenuation of brain derived neurotrophic factor (BDNF) by ethanol and cytoprotective effect of exogenous BDNF against ethanol damage in neuronal cells. J Neural Transm 112: 1005–1013.
48. Finck BN, Kelly DP (2006) PGC-1 coactivators: inducible regulators of energy metabolism in health and disease. J Clin Invest 116: 615–622.
49. Castillo-Quan JI (2011) Parkin' control: regulation of PGC-1alpha through PARIS in Parkinson's disease. Dis Model Mech 4: 427–429.
50. Kranz K, Warnecke A, Lenarz T, Durisin M, Scheper V (2014) Phosphodiesterase type 4 inhibitor rolipram improves survival of spiral ganglion neurons in vitro. PLoS One 9: e92157.
51. Hulley P, Hartikka J, Abdel'Al S, Engels P, Buerki HR, et al. (1995) Inhibitors of type IV phosphodiesterases reduce the toxicity of MPTP in substantia nigra neurons in vivo. Eur J Neurosci 7: 2431–2440.
52. Lin MT, Beal MF (2006) Mitochondrial dysfunction and oxidative stress in neurodegenerative diseases. Nature 443: 787–795.
